# Laparoscopic versus open left lateral segmentectomy

**DOI:** 10.1186/1471-2482-9-14

**Published:** 2009-09-07

**Authors:** Kirstin A Carswell, Filippos G Sagias, Beth Murgatroyd, Mohamed Rela, Nigel Heaton, Ameet G Patel

**Affiliations:** 1 Institute of Liver Studies, King's College Hospital, London, UK; 2 Department of Gene and Cell-Based Therapy, King's College London, UK

## Abstract

**Background:**

Laparoscopic liver surgery is becoming increasingly common. This cohort study was designed to directly compare perioperative outcomes of the left lateral segmentectomy via laparoscopic and open approach.

**Methods:**

Between 2002 and 2006 43 left lateral segmentectomies were performed at King's College Hospital. Those excluded from analysis included previous liver resections, polycystic liver disease, liver cirrhosis and synchronous operations. Of 20 patients analysed, laparoscopic (n = 10) were compared with open left lateral segmentectomy (n = 10). Both groups had similar patient characteristics.

**Results:**

Morbidity rates were similar with no wound or chest infection in either group. The conversion rate was 10% (1/10). There was no difference in operating time between the groups (median time 220 minutes versus 179 minutes, p = 0.315). Surgical margins for all lesions were clear. Less postoperative opiate analgesics were required in the laparoscopic group (median 2 days versus 5 days, p = 0.005). The median postoperative in-hospital stay was less in the laparoscopic group (6 days vs 9 days, p = 0.005). There was no mortality.

**Conclusion:**

Laparoscopic left lateral segmentectomy is safe and feasible. Laparoscopic patients may benefit from requiring less postoperative opiate analgesia and a shorter post-operative in-hospital stay.

## Background

Laparoscopic liver surgery, first performed in 1992 [1], is becoming the method of choice as surgical expertise in advanced laparoscopic techniques has developed. Laparoscopic enthusiasts have shown that it is safe and feasible to perform laparoscopic liver surgery [2-5]. Due to its anatomical accessibility left lateral segmentectomy (LLS) has been considered the training operation for all liver surgeons [6].

Proposed benefits of laparoscopic liver surgery include reduced overall blood loss, shorter hospital stay and less post-operative pain with a faster return to normal activity. But there are concerns as reported complications have included compromised oncological integrity [7-11], uncontrollable bleeding [10,12,13] and gas emboli [14-17].

To date one study has compared laparoscopic left lateral segmentectomy with an open approach using historical case controls [18]. Findings confirmed that the laparoscopic approach was safe and feasible, yet had significantly longer operating times and no difference in post-operative in-patient stay. The aim of this study was to undertake a contemporaneous comparison between laparoscopic and open left lateral segmentectomies.

## Methods

**Figure 1:**
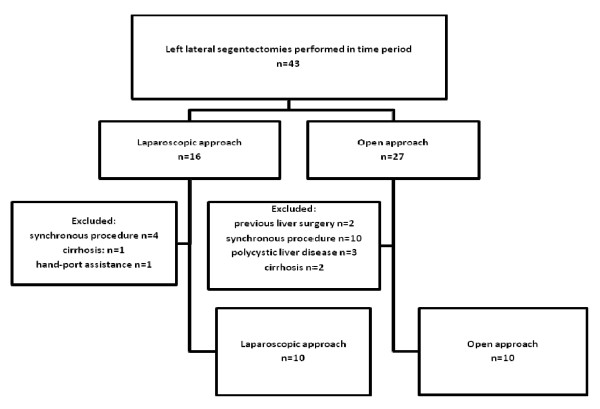
**Attrition diagram**.

We undertook a retrospective cohort study of the left lateral segmentectomies in our institution between July 2002 and October 2006 (n = 43). Cases were included on an intention to treat basis however, in an attempt to reduce bias, patients having previous liver resections (n = 2), synchronous operations (n = 14), polycystic liver disease (n = 3), liver cirrhosis (n = 3) and hand port assisted procedure (n = 1) were excluded (see figure 1). This resulted in 20 left lateral segmentectomies for comparison, 10 in the laparoscopic (LG) and 10 in the open group (OG). Selection was based on referral to the individual consultants with all laparoscopic operations performed by a single surgeon (AGP) and open operations under the care of two surgeons (NH, MR). Selection-bias was minimised by the random referral policy to the individual surgeons over this time period. All cases were discussed at the liver multi-disciplinary meeting pre-operatively. A detailed review of the medical records was conducted. Data collection included patient characteristics, site of lesion, operative details, postoperative analgesic requirements, morbidity and mortality, postoperative in-hospital stay, pathology of specimen, weight of resected specimen and tumour clearance margins. Ethical approval was not required.

All patients were operated on in the supine position under general anaesthesia with endo-tracheal intubation. A broad spectrum antibiotic (Tazocin, Wyeth Laboratories, Madison NJ) was given to all patients. Prophylaxis against deep vein thrombosis was given in the form of low-molecular weight heparin, thrombo-embolic deterrent stockings and intra-operative intermittent pneumatic compression boots. Staging laparoscopy with intra-operative ultrasound was performed to exclude peritoneal disease and to identify any additional tumours, as appropriate.

### Laparoscopic technique

With the surgeon standing to the right of the patient pneumoperitoneum was established after accessing the abdominal cavity using an open Hasson technique.

**Figure 2:**
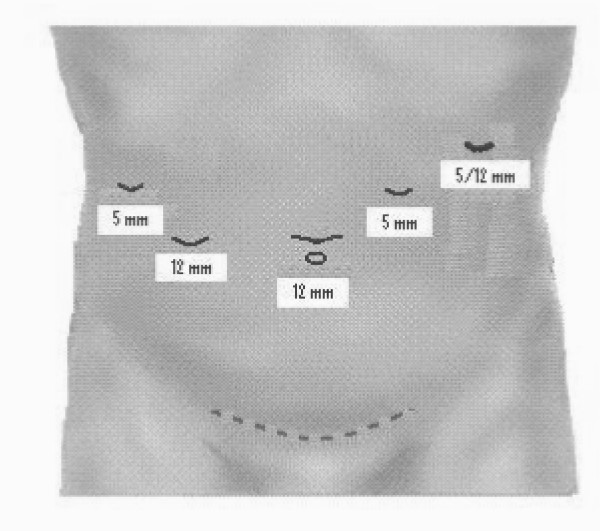
**Trocar placement for laparoscopic left lateral segmentectomy**. For extraction a pfannenstiel incision is usually used.

The initial port location varied depending upon previous surgery. Intra-abdominal pressure was kept at approximately 15 mmHg. Four additional ports were inserted (figure 2). A 30° laparoscope was used. The falciform and left triangular ligament was divided using a harmonic scalpel (Ethicon, Endo-Surgery Inc. Cincinnati Ohio). The falciform ligament was used to retract and manipulate the left lobe of the liver.

The line of resection was marked using the diathermy hook, 5 mm left of the falciform ligament and transected using a harmonic scalpel. In the umbilical fissure to the left of the falciform ligament the segment II/III pedicles were stapled and divided using Endo-GIA, 30 mm vascular staples (US Surgical, Norwalk CT). Liver parenchyma was transected using a harmonic scalpel +/- .Cavitron Ultrasonic Surgical Aspirator (CUSA^®^, Valleylab, Boulder CO). The left hepatic vein was approached intra-parenchymally and transected with Endo-GIA 30 mm vascular staples. The final attachment to the left triangular ligament close to the diaphragm was divided using hook diathermy and the specimen freed. Specimens were removed using a retrieval bag through a pfannenstiel or low midline incision. Fibrin glue was applied to the surface of the liver if required and a closed suction drain was placed near the transected liver. The skin was closed after infiltration of local anaesthetic (0.5% bupivacaine).

### Open technique

The open left lateral segmentectomies were performed as described by Bismuth [19].

A laparotomy was performed via a transverse subcostal incision with a midline extension if required. The left lobe of the liver was mobilised and the parenchyma was transected using CUSA^® ^and argon beam coagulation. With full mobilisation of the left lobe of the liver, the segment II/III pedicles were ligated and divided. The left hepatic vein was clamped at the end of the parenchymal transection and sutured with 5/0 prolene. Transection occurred through the liver parenchyma using CUSA^® ^and argon. Haemostasis was assured with the use of fibrin glue, and a drain was inserted.

### Data Analysis

Statistical analysis was performed using Mann-Whitney *U *test unless otherwise stated. P-values < 0.05 were considered to be statistically significant. All data is reported as median (range).

## Results

Of the 43 liver resections performed in our institution between July 2002 and October 2006, 20 patients met the inclusion criteria for this study with 10 laparoscopic and 10 open left lateral segmentectomies. Patients presented with either solid lesions, symptomatic liver cysts or liver abscesses, within segments II/III.

There were no differences in the patient characteristics between the two groups (Table 1). Median patient age was 54 years (25 - 72). 50% of patients were men (n = 10). The patients had similar American Society of Anaesthesiology (ASA) grading (I:II:III); 1:5:4 in the laparoscopic group and 2:6:2 in the open group. Indication for left lateral segmentectomy was malignant lesions in 50% of cases (n = 10).

**Table 1:** Patient Characteristics

	**Laparoscopic n = 10**	**Open n = 10**
**Median age in years (range)**	55 (34 - 72)	56 (25 - 68)
**Gender**		
Male	5	5
Female	5	5
**ASA Grade (I:II:III)**	1:5:4	2:6:2
**Diagnosis**		
Malignant	6	4
Benign	4	6

Two patients in each group (20%) developed early complications (< 30 days postoperatively). Minor morbidity in the laparoscopic group (LG) included one urinary tract infection, and a haematemesis (possibly secondary to non-steroidal anti-inflammatory drugs) which settled conservatively. In the open group (OG) one patient developed supraventricular tachycardia four days post-operatively, exacerbated by low potassium levels, which resolved spontaneously. The second patient developed hypoxia due to atelectasis, pleural effusions and a superficial haematoma. Only one late complication occurred, a small incisional hernia at the junction of the Mercedes incision in the OG 17 months postoperatively. No statistically significant difference in morbidity between the two groups was found (p = 0.725). There was no surgical mortality in either group.

In the laparoscopic group one conversion to open (10%) was necessary and occurred early in the series. The LLS was performed for a liver tumour that showed evidence of recent bleeding on a computerised tomography (CT) scan. Intra-operatively the tumour/haematoma from the left lateral segment was found adherent to the greater curve of the stomach, which required a wedge resection of the stomach. In order to avoid narrowing of the oesophago-gastric junction the procedure was completed via an open approach.

Portal triad clamping was not used in the laparoscopic approach. In the OG it was used intermittently in 50% (5/10) of patients, consultant preference. The median cumulative clamp time duration was 35 minutes (20-60). There was no significant difference in post-operative AST changes between the LG, OG (portal triad clamping) and OG (no portal triad clamping), one-way ANOVA. Within the LG one patient required an intra-operative blood transfusion (3 units) whilst in the OG two patients, both with liver abscesses, required a transfusion (1-2 units). There was no statistical difference in intra-operative blood transfusion requirement between the two groups (p = 0.782).

The median operating time for the LG was 220 minutes (116-335 minutes) versus 179 minutes (118-229 minutes) for the OG. No statistically significant difference was found between the two groups, p = 0.315 (see Table 2). The laparoscopic operating time has reduced over the study period with median operating time of 240 minutes in 2002, reduced to 163 minutes in 2006.

**Table 2:** Intra- and post-operative outcomes

	**Laparoscopic** **n = 10**	**Open** **n = 10**	**p-value**
**Intra-operative**			
Operating time (mins) (range)	220 (116-335)	179 (118-229)	p = 0.315
Clamping time (mins)	0	35 (n = 5)	
Blood Transfusion (%)	10	20	p = 0.782
**Post-operative**			
In hospital stay (days)	6	9	p = 0.005
Epidural use	1	9	p = 0.003
Opiod use (days) (range)	2 (1-5)	5 (2-14)	p = 0.005
Level II care stay (%)	80	100	p = 0.282
Weight of specimen (g) (range)	243.5 (99-577)	439 (213-1480)	p = 0.023
Resection margin (mm) (range)	15 (3-30)	14.5 (0->20)	p = 0.669

The LG had less analgesic requirements than the OG. This was exemplified by the statistically significant median postoperative opiate use 2 days (1-5) in LG versus 5 days (2-14) in the OG (p = 0.005).

Within the LG 80% (n = 8) of patients required one night in level II care. In the OG 100% of the patients spent one night in level II care (p = 0.282), with one patient requiring 4 nights of level II care. This patient was admitted as an emergency with a liver abscess in the left lateral segment of the liver. The prolonged level II care stay was due to multiple factors: poor post-operative analgesic control (unilateral epidural block); ischaemic changes on ECG (Troponin I negative) and a hypoxic episode day 2 post-operatively. A subsequent CT pulmonary angiogram revealed no pulmonary emboli.

The median postoperative in-hospital stay for the LG was 6 days (2-7) compared to 9 days (6-14) for the OG, showing a highly significant difference, p = 0.005 (see Table 2).

Histology is shown in Table 3. 60% (n = 6) of lesions in the LG and 40% (n = 4) in the open group were malignant. The median total weight for the resected specimens in the LG was 243.5 g (99 g-577 g) versus 439 g (213 g-1480 g) in the OG (p = 0.023). Resection margins for all malignant lesions were clear. In the LG the median resection margin was 15 mm (3-30 mm) with the median in the OG 14.5 mm (< 1 mm ->20 mm)(p = 0.669).

**Table 3:** Pathology

**Patient no**.	**Approach (laparoscopic/open)**	**Pedicle clamping (yes/no)**	**Histology**	**Size of lesions (cm)**	**Resection margin (mm)**	**Specimen weight (grams)**
1	Laparoscopic	No	Simple cysts	0.17 with minute satellite cysts	n/a	325
2	Laparoscopic	No	FNH	7 × 6 × 6	Clear	150
3	Laparoscopic	No	FNH	7.5 × 7 × 5	Clear	452
4	Laparoscopic	No	Simple cysts	Multiloculated 0.5-14 diameter	N/a	175
5	Laparoscopic	No	Colorectal metastases	3.5 × 3.2 × 3.0	20	225
6	Laparoscopic	No	Colorectal metastases	3.2 × 1.5 × 2.0	10	269
7	Laparoscopic	No	Colorectal metastases	8 × 10 × 9	3	577
8	Laparoscopic	No	Colorectal metastases	No information	4	248
9	Laparoscopic	No	Colorectal metastases	1 × 1 × 0.4	30	239
10	Laparoscopic	No	Colorectal metastases	2.3 × 2.0 × 1.2	23	99
11	Open	No	Abscess	3.0 and 4.0	n/a	450
12	Open	No	Abscess	14 × 7 × 6	20	680
13	Open	Yes	Leiomyosarcoma	14 × 18 × 7	17	1480
14	Open	No	HCC	15 × 11 × 11	12	975
15	Open	No	FNH	1.2 × 1.5 × 1.8	16	350
16	Open	Yes	Hydatid cyst	~3.0 diameter	0	671
17	Open	Yes	FNH	9.8 at greatest diameter	0	428
18	Open	No	Colorectal metastases	8 × 6 × 4.5	> 20	213
19	Open	Yes	Abscess	5 × 5 × 3.5	3	278
20	Open	Yes	Colorectal metastases	1.7 at greatest diameter	0	265

The median follow-up in the LG was 18 months (0-63) and 6 months (0-33) in the OG. Of the malignant cases, post-LLS recurrence in the liver has occurred in 2/6 of the LG (median 14 months) and in 2/4 of the OG (19 months [10-28]). To date no port-site metastases have occurred in these patients.

## Discussion

This study echoes the growing body of evidence demonstrating that a laparoscopic approach to liver surgery provides tangible benefits to both patient and hospital. As surgical skill develops it is anticipated that left lateral segmentectomy will shift from being a traditionally open procedure to a laparoscopic one. This in turn may benefit an increasing number of patients for whom open surgery could be considered high risk.

The findings of this study are consistent with other published series showing laparoscopic liver surgery to be feasible and safe [2-5,12,20]. A 20% post-operative morbidity rate (< 30 days) was comparable between the open and laparoscopic group. There were no liver related complications, chest or wound infections in either group.

Within the laparoscopic group, conversion to an open procedure occurred in one patient (10%) to ensure narrowing of the oesophago-gastric junction did not occur. The need to convert to an open procedure has commonly resulted from uncontrollable bleeding [12,13,18,20]. As surgical techniques improve conversion rates have decreased, with early experiences reporting a 33% conversion rate [13] to Chang et al [20] reporting a 2.7% conversion rate due to bleeding. In this series no operations were converted to open due to bleeding.

During design of this study we excluded cases in both groups which had undergone previous liver resections, synchronous operations (liver resections, biliary procedures, reversal of ileostomy, hernia repairs), polycystic liver disease (inc. fenestration of liver cysts), liver cirrhosis and hand-port assisted procedures. The main objectives of this study were to compare operative time, analgesic requirement and morbidity between the open and laparoscopic approach. As such we felt it necessary to control for these variables despite its impact on sample size. This is a retrospective cohort study and as such we recognise the slight disparity in heterogeneity of pathologies included in the final analysis.

Portal clamping was not required in the LG. In the OG 50% (n = 5) of patients underwent clamping for a median duration of 35 mins (11-60 mins). Early experiences in the literature utilised portal clamping to a greater extent with the laparoscopic versus the open approach[18], with a resultant decrease in blood loss. In this series, the open group maintained a low central venous pressure (CVP) and utilised intermittent portal clamping resulting in minimal need for blood transfusion (20% n = 2). Whilst not requiring portal clamping, the laparoscopic technique relied on the positive pressure of the pneumoperitoneum, which in turn minimised potential blood loss, with only one patient (10%) receiving a blood transfusion.

In laparoscopic liver surgery there are long standing concerns regarding gas emboli [14] with laparoscopic surgeons opting for abdominal wall lifting (gasless laparoscopy) or using low CO_2 _pressures to maintain pneumoperitoneum, to minimise any potential risk. Animal studies have shown an increased risk of cardiac arrhythmias [15] and gas emboli in those with 16 mmHg compared to 8 mmHg (after the left hepatic vein was left open for 3 minutes) [16]. Whilst this implies increased pneumoperitoneal pressures may exacerbate the risk of gas emboli, no human data exists. Potential advantages of increased pneumoperitoneal pressure include reduction in blood loss and improved visualisation of the operative field. In this series pneumoperitoneal pressures were maintained at 15-20 mmHg with no clinical adverse incidents however, a prospective study in this field is overdue.

No significant difference was found in the operating time between the two groups (220 vs 179 minutes, p = 0.315), consistent with both Mala et al [21] and Mamada et al [22]. Other groups have shown longer operating times in the LG [18,23]. Of interest, in the LG and the OG the shortest operating times were comparable (116 vs 118 minutes). It must be highlighted that the left lateral segmentectomy is considered a training operation. As such, surgeons in training (under the supervision of the Consultant) operated on some of the open group (n = 4). These data also includes the first laparoscopic LLS performed by the laparoscopic surgeon therefore reflecting the learning curve; the median laparoscopic operating time in 2002 was 240 minutes and by 2006 it was 163 minutes. It is expected that laparoscopic operating time for this procedure will continue to reduce however, these factors may affect comparison of operative time.

One patient in the LG had an epidural (10%), while 90% (n = 9) required an epidural in the OG (p = 0.003). It is not routine for the laparoscopic liver patients to require epidural anaesthesia but is protocol for open liver resections to have an epidural inserted preoperatively. In the LG, port-site infiltration of local anaesthetic was used to optimise post-operative analgesia however, port-site infiltration has been shown to have no impact on post-operative pain after analgesia [24]. To account for these discrepancies, we assessed the total number of days post-operative opiate analgesia was required, including all methods of opiate administration. This revealed significantly less opiate analgesia was required in the LG postoperatively (2 vs 5 days, p = 0.005). In their randomised clinical trial Veldkamp et al [25] found there was a need for fewer analgesics in the laparoscopic vs open group following surgery for colon cancer. This reduced demand for post-operative analgesia was also found by Farges et al [26] with 50% less morphine (15.5 mg vs 31.6 mg) administered in the laparoscopic versus open approach following liver resection.

All patients in the OG and 80% of the LG were routinely transferred to level II care with a median stay of one night. With increased confidence and experience this policy has since been modified. Currently patients from both groups no longer routinely require level II care, and the more stringent use of these resources has favourable cost implications for future service development.

In this study the median post-operative in-hospital stay was significantly less in the LG than OG (6 vs 9 days, p = 0.005). In other comparative studies, laparoscopic versus open liver resections for colorectal metastases the median post-operative stay was 4 days vs 8.5 days (p < 0.001) [13], and for hepatic resections 7.8 days vs 11.6 days (p < 0.05) [23] and 10.4 vs 18.0 days (p < 0.05) [21]. This demonstrates reduced post-operative in-patient stay is a reproducible, safe benefit of laparoscopic liver resection.

The weights of the resected specimens in the LG were significantly lower than the OG (p = 0.023). On histological examination of the normal liver in the specimens there was no difference with respect to clamping to account for the weight disparity. Mala et al [21] also noted significantly lighter specimens resected laparoscopically, along with no significant difference in resection margin involvement. This may reflect a slight difference in operative techniques between the two approaches. The left hepatic vein is approached and transection of the major vessels occurs intra-hepatically in the laparoscopic group. We speculate that this results in a rounded superior resection, accounting for this difference.

Oncological integrity is often questioned in laparoscopic liver resections for malignancy. In this study resection margins were clear in all malignant cases (median laparoscopic 15 mm vs open 14.5 mm). However, early experiences of laparoscopic oncological surgery resulted in an increased fear of developing abdominal wall metastases after laparoscopy for hepatic cancer compared with open surgery. Hypotheses suggested the peritoneum may be damaged by the pneumoperitoneum, inducing intra-peritoneal tumour growth [11]. However this notion is becoming outdated. A short-term animal study by Agnosti et al [27] showed no significant difference in terms of tumour growth, irrespective of gas or pneumoperitoneum pressure used. Jacobi et al [28] suggest intra-peritoneal tumour growth increases for pressures < 10 mmHg and decreases at higher pressures. There is also further evidence that reported tumour growth in colonic cancer was significantly reduced after CO_2 _laparoscopy when compared to gasless laparoscopy [29]. A meta-analysis of over 1 500 patients undergoing laparoscopic vs open colectomy for colon cancer found no difference in the 3 year survival rate (82.2% vs 83.3%) [30], concluding that a laparoscopic approach is indeed oncologically safe.

Any potential risk of port-site metastases can be reduced by maintaining an intact surgical specimen and using plastic retrieval devices [20,31], thus minimising contact with the extra-peritoneal structures.

There is evidence that the reduced stress response of laparoscopic surgery may be preferential in the malignant cases due to associated lower rates of infection and potential reduction in tumour recurrence [32,33]. Animal data suggests that increased surgical stress augments cancer metastasis via surgical stress-induced expression of proteinases in the target organ of metastasis [34]. There is also a diminished stress response to the laparoscopic approach versus open liver resection, preserving immune function [32].

An additional benefit of the laparoscopic approach is reduced adhesion formation [32] which may facilitate further liver resections for metastases. With an increasing trend for non-anatomical and segmental resections with increased parenchymal preservation [35], repeat metastectomies (and re-resections) are becoming more common. Petrowsky et al [36] report similar outcomes for patients having either a primary or repeat laparoscopic resection following an initial resection performed laparoscopically. These findings have led to an increased number of patients undergoing further liver metastatectomy, presenting a further interventional option as multiple staged liver resections become more commonplace.

## Conclusion

The laparoscopic approach to left lateral segmentectomy is safe and feasible with reproducible results. In a specialised unit, it may offer no difference in operating time, morbidity and mortality rates and oncological clearance. Potential benefits include reduced opiate analgesic requirements and shorter hospital stay however, the importance of patient selection cannot be over-emphasised.

## Abbreviations

ASA: American Society of Anaesthesiology; CT: computerised tomography; CUSA^®^: Cavitron Ultrasonic Surgical Aspirator; CVP: central venous pressure; FNH: focal nodular hyperplasia; HCC: hepatocellular carcinoma; LG: laparoscopic group; LLS: left lateral segmentectomy; OG: open group.

## Completing interests

The authors declare that they have no completing interests.

## Authors' contributions

KC conceived of the study, participated in its design, data collection and analysis and drafted the manuscript. FS participated in its design, data analysis and drafting of the manuscript. BM assisted with data collection and drafting of the manuscript. MR and NH participated in study design, carried out some of the operative procedures, assisted with data collection and manuscript revisions. AGP participated in study design and coordination, carried out the laparoscopic procedures, assisted with data collection and supervised manuscript revisions.

All authors read and approved the final manuscript.

## Pre-publication history

The pre-publication history for this paper can be accessed here:


